# Musculoskeletal Risks: RULA Bibliometric Review

**DOI:** 10.3390/ijerph17124354

**Published:** 2020-06-17

**Authors:** Marta Gómez-Galán, Ángel-Jesús Callejón-Ferre, José Pérez-Alonso, Manuel Díaz-Pérez, Jesús-Antonio Carrillo-Castrillo

**Affiliations:** 1 Department of Engineering, University of Almería, Research Center CIMEDES (CeiA3), 04120 Almería, Spain; mgg492@ual.es (M.G.-G.); jpalonso@ual.es (J.P.-A.); madiaz@ual.es (M.D.-P.); 2 Laboratory-Observatory Andalusian Working Conditions in the Agricultural Sector (LASA), 41092 Seville, Spain; 3 School of Industrial Engineering, University of Seville, 41092 Seville, Spain; jcarrillo3@us.es

**Keywords:** biomechanics, musculoskeletal disorders, RULA, ergonomics, applications

## Abstract

The objective of this study was to reveal RULA method applications in terms of the knowledge, country, year and journal categories. The search was performed using the “Web of Science Core Collection”. The period from 1993 to April 2019 was selected. Eight hundred nine results were obtained, of which 226 were used. The largest number of publications was determined to be in the fields of industry and health and social assistance, which coincides with the OWAS and Standardized Nordic Questionnaire methods. By country, the USA stands out for its greater number of research studies and categories that are encompassed. By date, 2016 was the year when more studies were carried out, again coinciding with the Standardized Nordic Questionnaire. By journal, “Work—A Journal of Prevention Assessment and Rehabilitation” is highlighted, as it is for the REBA method as well. It was concluded that RULA can be applied to workers in different fields, usually in combination with other methods, while technological advancement provides benefits for its application.

## 1. Introduction

### 1.1. Musculoskeletal Disorders (MSD)

One of the most common work diseases in Europe are musculoskeletal disorders. These appear in various areas of the body, the most common developing in the back and upper extremities [1].

Among the causes that stand out for their appearance are the physical (manual, forced or frequently repeated movements, harmful postures and vibrations, etc.) and those relating to work organisation (high work rate, schedule, routine work, etc.) [2].

This type of disorder has numerous consequences for the affected worker, but also for businesses and countries (at the economic level) [3].

To prevent this occupational disease, it is necessary to identify all the risk factors that occur during work. Once determined, preventive measures should be taken to avert them, or actions taken to reduce them [2]. Some authors propose measures such as rotating workers between different jobs [4], providing ergonomic training to workers [5], designing ergonomic tools for MSD analysis in the workplace [6], redesigning work equipment from the ergonomic perspective [7], etc.

### 1.2. Assessment Methods

There are numerous methods for assessing musculoskeletal disorders. These can be classified into three main groups: direct, semi-direct and indirect (Table 1 [8]).

Of the three groups above, the most economical methods are the indirect ones since they are based solely on completing questionnaires. The opposite is true in the other two cases as software licenses are required and sensors have to be purchased. Direct methods are the most accurate because they provide virtually automatic information. In terms of complexity, direct and indirect methods stand out—in the former because the use of sensors can be a nuisance for the worker and, in the latter, because of the subsequent statistical analysis [8].

When selecting a method for a particular study, it is advisable to consider several criteria (cost, accuracy, complexity, application time, etc.) and to analyse the advantages and disadvantages of each.

### 1.3. Examples of Direct, Semi-Direct and Indirect Methods

**Figure 1:**
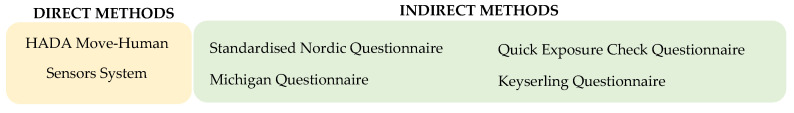
Examples of direct and indirect methods.

**Figure 2:**
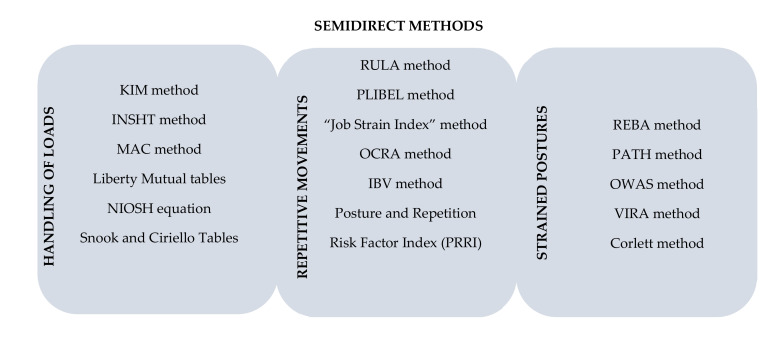
Examples of semi-direct methods (see Appendix A).

The following are some examples of direct, semi-direct, and indirect assessment methods (Figure 1; Figure 2; [9,10,11,12,13]; [14,15,16,17,18,19,20,21,22,23,24,25,26,27,28,29,30]).

### 1.4. Rapid Upper Limb Assessment (RULA) Method

The RULA method was designed in 1993 by McAtamney and Corlett. Its objective is to know if workers are exposed to MSD risk factors in the upper extremities during the performance of their work [20].

**Figure 3:**
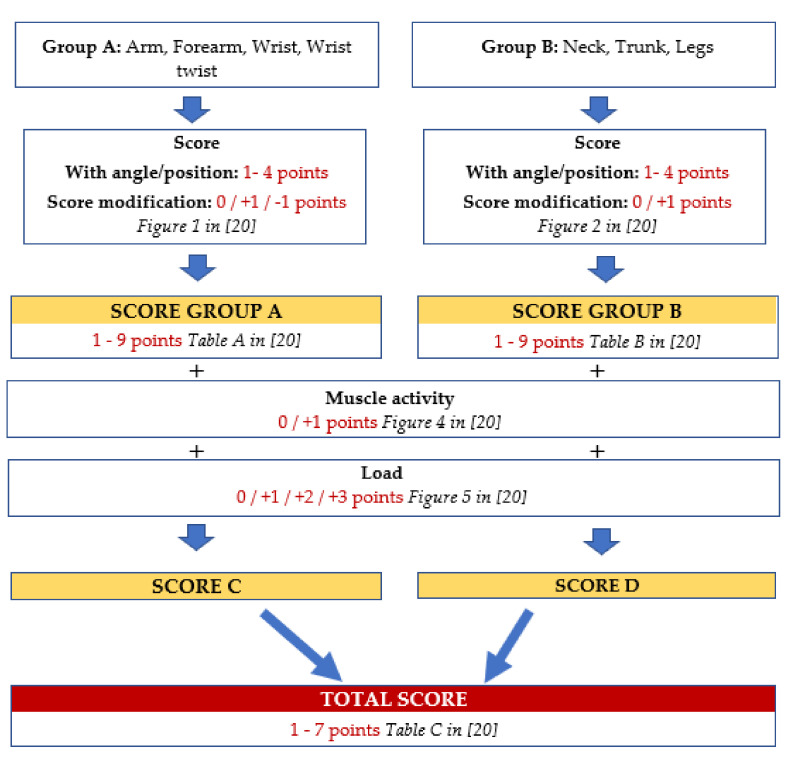
RULA Scores [20,31].

The method assesses three factors: the posture of the different areas of the body, the load or force exerted and the muscle activity (static posture or repetitive movements). The body regions which RULA focuses on are divided into two groups [31]:

▪ Group A: arm, forearm, wrist and wrist turn.
▪ Group B: neck, torso and legs.
▪ Three main stages [20,31] are performed to carry out the assessment using this method:
▪ Observation of postures. This is done while the worker performs the task and can be realised in three ways: direct observation or by taking images or videos. The postures to be assessed (those most repeated, those performed for more than 10% or 15% of the task and those that are most harmful) are selected. Only one side of the body is analysed, namely the one which suffers most harm; however, if they are very different, then both sides are assessed.
▪ Scores. For the postures to be assessed, the angle must be measured between each of the body areas and the vertical. The lower extremities are not measured, although it is taken into account whether the posture is balanced and supported. Using these data, which are modified by different criteria and considering the load factors and muscle activity, the RULA scores are calculated (Figure 3).
▪ Action levels. From the final scores, one obtains the action level. RULA differentiates four levels:
  ⮚ Level 1 (Score: 1–2). No action is needed.
  ⮚ Level 2 (Score: 3–4). Measures should be taken, but not in the short term.
  ⮚ Level 3 (Score 5–6). Measures should be taken in the short term.
  ⮚ Level 4 (Score 7). Urgent action must be taken.

#### 1.4.1. Advantages

Some advantages of the RULA method include:

▪ It is a reliable method to use for repetitive tasks, mainly in the upper limbs [31].
▪ It has been applied to workers across very different areas [31].
▪ The assessor needs no experience in order to apply it during the observation phase [20].
▪ It is not very complex to apply. The method is simple to use [32].
▪ It can be applied with the help of software [31,32].

#### 1.4.2. Limitations

Among the disadvantages of the RULA method are the following:

▪ It results in a high-level risk for non-permanent jobs [31].
▪ The left and right side of the body are assessed independently [32].
▪ It does not take into account the time the worker takes to carry out the task [32].

### 1.5. Objective

The aim of this work is to review all the studies in which the RULA method has been applied from 1993 to April 2019 and to analyse them according to the following categories: knowledge, country, year and journal.

## 2. Materials and Methods

To search for information about the different RULA method applications, the University of Almería’s library website was accessed. There, we used the “Web of Science” (WoS) database, the license of which is managed by the Spanish Foundation for Science and Technology (FECYT).

The search was done by selecting the “Web of Science Core Collection” and the advanced search option, in which “so = Applied Ergonomics” was introduced. The “Create Citation Reports” option was selected. In this way, the total number of available citations was consulted for the original article proposing the RULA method by McAtamney and Corlett [20].

The number of studies obtained was 809, in the period from 1993 to 29 April 2019. Of these, using the title and abstract of each study, we finally selected a total of 226 results, discarding those that did not include the method’s application. The final studies were from 1998 to 2019.

It should be noted that the main limitation of the information search is that we only used the Web of Science Core Collection; no other databases were accessed. Therefore, some studies may have been overlooked with the search method used.

## 3. Results and Discussion

The studies obtained from the search have been analysed and classified according to the categories of knowledge, location (countries), publication date (years) and journals.

### 3.1. Classification by Knowledge Categories

A particular classification [33] has been used to order the RULA studies according to the knowledge categories. Table 2 shows the different areas presented, the designated nomenclature and the number of published studies.

In total, 19 categories are differentiated. One can observe that “Manufacturing” stands out as having more studies than the rest, a total of 74. This is followed by 38 and 25 publications corresponding to “Human health and social work activities” and “Other areas not previously included “, respectively (Table 2).

In contrast, two fields are differentiated in which only one research study is recorded—“Water supply, sewerage, waste management and remediation activities” and “Financial and insurance activities” (Table 2).

**Figure 4:**
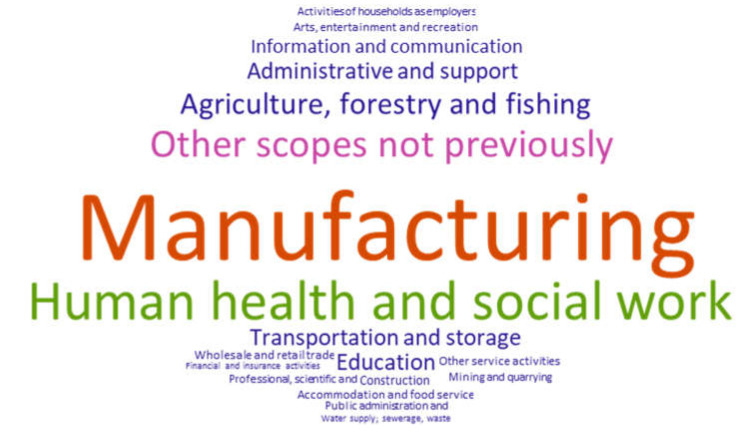
Knowledge categories and frequency.

Figure 4 shows the different areas of knowledge based on the frequency of the published studies.

Below is a description of each of these categories [33].

#### 3.1.1. Agriculture, Forestry and Fishing

Table 3 summarises the studies related to this category.

##### Crop and Animal Production, Hunting and Related Service Activities

The RULA method has been used for the ergonomic assessment of farm workers working on various crops or specific tasks.

In fruit crops, Thetkathuek et al. [38] applied it together with the Nordic Musculoskeletal Questionnaire and a tasks checklist. In total, 861 farm workers took part in Thailand. They showed that men with more than 10 years of experience had their necks most affected and women their lower backs. In blueberry harvesting, Kim et al. [36] used it in conjunction with the Borg CR10 scale, electromyography, the Cumulative Trauma Disorders (CTD) index and NIOSH (National Occupational Institute for Safety and Health). They concluded that the risk was reduced by carrying out the work with the help of machinery.

Three studies were carried out on oil palm harvesting. In two of them, other ergonomic tools were also used. In the first study, 109 workers were assessed, while three and seven were assessed in the other two. The results for all three showed that most workers were exposed to a very high level of risk when carrying out the tasks. Changes were required as a matter of urgency [46,47,48].

Three other studies were conducted into rice cultivation. Pal and Dhara [37] studied 166 workers in India. They also used other methods such as REBA (The Rapid Entire Body Assessment), OWAS (The Ovako Working Analysis System), etc. The results showed the frequent appearance of MSD, with the most affected areas being the lumbar, hip, wrist, shoulder and knee regions. In the other two studies, farm workers were assessed when using machinery during the tasks: in one case using threshing machines and in the other using a cultivator. For both, it was concluded that there was a high risk of MSD [44,49].

In olive cultivation, Pardo-Ferreira et al. [40] assessed farm workers performing pruning with a chainsaw. Other methods were also used, in particular REBA and OWAS. They determined that change was needed, as workers were exposed to ergonomic risks.

Another series of studies was conducted using the RULA method to assess different tasks. Jain et al. [39,41] carried out two studies focusing on manual tasks, also using a questionnaire. The first concluded the frequent development of musculoskeletal disorders in workers; this coincided with the second, which indicated a score (RULA) of 5 or above for 92% of the workers.

Vazquez-Cabrera [45] investigated farm workers during crop stringing. They simulated different ways to do it in a laboratory according to the height, crop weight and guides used. Among other findings, heights of 1.4 m were shown to be acceptable, with 1.2 and 1.6 m heights also being possible. Weights over 2 kg were found not to be suitable.

Finally, some authors set out specific objectives. Kong et al. [42] used RULA to assess Korean farm workers as they adopted 96 postures. The aim was to apply RULA along with the REBA, OWAS and ALLA (Agricultural Lower Limb Assessment) methods in order to compare the last one with the previous three, proving it to be the most correct. Di Gironimo et al. [50] set out to redesign and improve the ergonomics of an agricultural tractor’s driver space. In addition to RULA, this was done using Catia V5 software (as well as other ergonomic tools) to design a 3D model. Some devices in the cab needed to be corrected to provide better ergonomics.

##### Forestry and Logging

Studies in this category using the RULA method have only been carried out in forestry.

Several authors focused on rubber harvesting workers (tappers). Meksawi et al. [51] also used a survey and a form. According to the RULA results, an action Level 3 was obtained, so changes needed to be made to the work. Pramchoo et al. [35] assessed the ergonomics of a new knife designed for tapping. Two groups participated, one of them using the new knife. In each group, half of the workers suffered discomfort related to the carpal tunnel syndrome. The Boston Carpal Tunnel Syndrome Questionnaire (BCTQ) was also applied. The new knife, among other advantages, reduced the discomfort from carpal tunnel syndrome in sufferers and improved the wrist posture.

Regarding forestry activity, two studies were performed to assess workers. Cremasco et al. [34] focused on tasks performed with a wood-chipper; they also used REBA in their assessment. Unver-Okan et al. [43] focused on forestry nurseries; they also employed REBA, OWAS and QEC (Quick Exposure Check). In both cases, it was determined that the RULA method was the most suitable for such assessments.

#### 3.1.2. Mining and Quarrying

Table 4 presents the only two studies related to this category.

In this field of knowledge, there are only two studies available in which the RULA method was applied—one for the ergonomic assessment of mine workers and the other, together with the REBA and OCRA (Occupational Repetitive Action) methods, in stone-carving workers. Both studies concluded that very high risks existed that required immediate changes [52,53].

#### 3.1.3. Manufacturing

Table 5 sets out the different publications available in the field of manufacturing.

##### Manufacture of Motor Vehicles, Trailers and Semi-Trailers

The RULA method was used in four research studies to ergonomically assess automotive workers. Each focused on various tasks such as painting, assembly and others. One of the studies used a human digital model, obtaining some benefits from its use [124]. In the others, in addition to the RULA method, other assessment tools were applied. It was concluded that the risk of musculoskeletal disorders existed. Two of them yielded high-level risks [55,75,123]. On the other hand, Mat et al. [63] used RULA to ergonomically assess a car seat. The seat was analysed after being designed with CATIA software and optimisation was performed. This showed a decrease in the level of risk.

##### Manufacture of Other Non-Metallic Mineral Products

Sain and Meena [56] used RULA, REBA and a questionnaire to perform an ergonomic analysis on brick furnace personnel. They concluded that some tasks needed to be modified as there was a risk of musculoskeletal disorders. Coinciding with this need to take action, Monteil et al. [115] employed RULA via a digital human model on roof slate manufacturing workers.

##### Manufacture of Fabricated Metal Products, Except Machinery and Equipment

The RULA method was used by Boulila et al. [57] along with REBA and a survey on milling, turning and drilling workers. Twelve postures were determined to negatively influence job performance. In relation to this, other authors redesigned a CNC milling machine to take worker ergonomics into account. They used RULA to analyse the postures assumed in the initial machine and those in the new design, with the latter being less harmful [90].

In addition, two studies found high risk levels according to the RULA method. The first was carried out on workers performing manual handling work in a metal stamping business. The Cornell Musculoskeletal Discomfort Questionnaire (CMDQ) [96] was also used. The second study focused on cutlery polishing workers [116].

Finally, some authors [126] studied the relationship between ergonomics at work and product quality in the metallurgical industry. RULA was used and, as a result, the work carried out was modified. Quality improvements were identified in the newly-produced products.

##### Food Product Manufacture

The RULA method was used in conjunction with REBA in the food industry. It was concluded that workers adopted postures that could lead to musculoskeletal disorders [65]. Similar results were obtained by applying RULA together with other assessment tools on pineapple peeling workers, obtaining a risk Level 3 assessment [76]. It was also applied in a sugar factory assessing the bag movement task. A high-risk level was determined [64].

In two other cases, negative results were shown for certain body regions. One of them applied the method to a frozen food business, along with other tools such as the Nordic Questionnaire. This was applied to both production and office workers. The greatest discomfort appeared at the elbow [77]. The other focused on workers in a meat packing factory. RULA, a human body diagram and anthropometric measurements were used. The shoulder was shown to be at risk. It was concluded that corrective measures were required [104].

Other authors used 3D simulation and the I-DEAS programme to replace the work of a robot (used for processing fruits and vegetables) with a worker. RULA and LBA (Lower Back Analysis) were employed for the ergonomic analysis. The results found a particular posture that the seated worker could adopt to avoid a high risk to the lower back and upper limbs [105].

Finally, a study was carried out on baristas tasked with tamping espresso coffee. RULA was used in conjunction with sensors placed on the column and a force plate. Lower scores were shown when using a flat tamper rather than a traditional one [58].

##### Manufacture of Computer, Electronic and Optical Products

According to RULA, assembly tasks in the electronics industry indicate high levels of risk for the workers who perform them. Two studies demonstrated this, the first applied the RULA method along with a questionnaire while carrying out an intervention to achieve more comfortable postures. The other supplemented it with REBA and the modified Nordic Questionnaire. Both agreed that the body areas most affected by MSD were the lumbar region and the wrists. However, one of them also included the hands and neck while the other included the shoulders [59,60].

Other authors set out to reduce neck discomfort in mobile phone assembly workers. To do this, they performed an ergonomic intervention. An assessment, using the RULA method and other tools, was carried out before and after the intervention. Improved worker ergonomics were achieved following the intervention [106,117].

Something similar was done during the manufacture of laptops. Materials were reused and two carts were created, one for refuse collection and the other for sorting and distributing labels. An assessment was carried out with RULA before and after using the new carts. Better ergonomic and work results were obtained [107].

##### Manufacture of Other Transport Equipment

The RULA method was used in three studies developed in the same year which looked into aircraft manufacturing. The purpose was to assess worker ergonomics. One of the studies also used the Strain Index demonstrating a relationship among work design, performance and ergonomics [66]. In another, RULA was used in conjunction with REBA to assess aircraft wing component assembly using immersive virtual reality to perform the task [67]. Finally, a maintenance work analysis tool was developed using CATIA software. RULA, OWAS and LBA [68] were employed together.

##### Manufacture of Machinery and Equipment N.E.C.

In this area, the RULA method was used in a single study. It was concluded that high-risk postures were present in pump assembly workers diving into wells [69].

##### Manufacture of Leather and Related Products

There has been repeated use of the RULA method for the postural assessment of shoe factory workers. In some studies, it has been combined with the OWAS method and in others with the Nordic Musculoskeletal Questionnaire. In all the cases studied, negative results were obtained for some of the postures adopted [70,78,92].

These conclusions coincided with those obtained in a study on manual shoe-sewing workers. The RULA method was used together with a questionnaire. The need for postural correction was demonstrated; in some cases, it was required immediately [91].

##### Manufacture of Basic Pharmaceutical Products and Pharmaceutical Preparations

Two studies were developed in the pharmaceutical industry using the RULA method and the Nordic Questionnaire. One concluded that RULA was not a very successful method for assessing this type of industry. The other aimed to assess lumbar and neck discomfort [71,79].

##### Textile Manufacture

In the textile industry, two research studies were carried out using the RULA method and questionnaires. Approximately 380 workers participated. The results concluded that MSD was common. One of the studies found that the body areas suffering the greatest discomfort were the neck, knees and lumbar region [81,97]. Using the same ergonomic tools, Dianat et al. [87] also established that the neck and back were the body regions most affected in sewing machine workers. They also pointed to other areas such as the shoulders, wrists and hands.

Other authors also employed RULA in the textile industry along with the Nordic Musculoskeletal Questionnaire, or an adaptation of it. They assessed 566 workers in one study and 283 in the other. Both studies concluded that high levels of risk were present in their results and therefore there was a need to take corrective actions, in some cases immediately. They agreed that the affected body areas included the torso, neck and arms, amongst others [108,113].

Other assessments were carried out in industries located in Indonesia and Cambodia. In the first of these, a virtual space with a human model was used and four tasks were analysed: cutting, sewing, putting on buttons and finishing. The Posture Evaluation Index (PEI) was used, which includes the LBA, OWAS and RULA scores. The second was performed using interviews and RULA. In both cases, workers could develop musculoskeletal disorders [83,114].

Analysis was also carried out on people working in rug-fixing workshops. They usually performed their tasks squatting. Using a survey, it was possible to deduce the most affected body regions, and then to design a new work posture from this information. This posture was subsequently assessed with RULA, demonstrating it to be less harmful [125].

Other authors found a way to organise clothing assembly tasks at particular workstations. To do this, they assessed both productivity and ergonomics; RULA was used for the latter [99].

Using the same ergonomic tools, Dianat et al. [80] concluded from the studies described above that there is a high onset rate of musculoskeletal disorders among sewing workers.

Finally, several ergonomic studies have also been carried out in the batik cap industry. One demonstrated the emergence of MSD among the workers, and the other two determined new work postures for specific tasks that were less harmful [82,88,98].

##### Manufacture of Basic Metals

Garcia-Garcia et al. [95] used RULA and REBA along with virtual simulation to assess workers in a metal factory. Kushwaha and Kane [74] utilised the method to ergonomically assess two crane cabs (the usual one and a new design) in the steel industry. The new cab was designed with CATIA software and was shown to improve worker ergonomics.

##### Manufacture of Chemicals and Chemical Products

The Nordic Musculoskeletal Symptoms Questionnaire (NMSQ) and the RULA method were used to study ergonomics in chemical industry workers when performing various tasks. RULA showed that they were suffering from MSD. Automation and work modification [73] were proposed as a solution.

##### Repair and Installation of Machinery and Equipment

The risks that occur during the maintenance of a REHM V8 furnace were analysed using a modification of the NTP 330 method. The ergonomic risks were also contemplated using the RULA method and interviews. It was concluded that the ergonomic risks, amongst others, were detrimental to workers [86].

##### Manufacture of Electrical Equipment

At a transformer factory in Brazil, worker ergonomics were studied using RULA and a questionnaire. Risk factors were shown to be related to the posture adopted and the work rate. Immediate changes to the tasks [93] were required.

##### Manufacture of Paper and Paper Products

CATIA P33 V5R14 software was used to recreate the posture adopted by workers in a packaging business. RULA was used for the ergonomic assessment. Certain harmful postures were determined, mainly in tasks where heavy loads were lifted, or tasks which included bending [100].

##### Manufacture of Rubber and Plastic Products

In total, 25 rubber sheet manufacturing workers were ergonomically assessed using RULA, REBA and OWAS, specifically looking at nine9 different tasks. Most of the postures adopted were repeated continuously and the job was not particularly comfortable for the workers. High scores for these working postures [109] were determined according to the methods applied.

##### Manufacture of Wood, and Wood and Cork Products, Except Furniture; Manufacture of Articles Made from Wicker and Plaiting Materials

Several research studies were conducted on sawmill workers, using RULA in conjunction with other assessment methods and ergonomic tools. The objective was to compare the results using the different methods to find if there was any agreement among them [118,120,121].

##### Other

This section includes studies that are also within the scope of industry, but which cannot be included in any of the classification groups used.

Most of these studies were carried out in the past 17 years. The first one performed aimed to establish the QEC method action categories. To do this, several industry jobs were analysed using QEC and RULA, and then the results were compared [127]. RULA was also compared to other methods (OWAS and REBA) by analysing 301 worker postures in industries such as the electronics, steel, chemical and automotive industries, as well as in a hospital. OWAS and REBA ranked 21% of the postures in Categories 3 and 4, while RULA ranked 56% of them [122]. This method was also compared to seven others by performing an ergonomic assessment of 567 industrial tasks. The results were shown to be different for the same work post depending on the method used. RULA determined a high risk for nearly 80% of the jobs [111].

Eswaramoorthi et al. [119] used RULA with CATIA V5 software to assess assembly line workers. This assessment led to changes in job design to reduce or eliminate waste, as these could cause an increase in the heavy physical burden on the worker. The method was also used in another assessment of this type, but in this case on a humanoid robot arm with similar postures to those adopted by a human. The goal was to assess the robot arm’s configurations [112]. Related to the above, a method was developed allowing one to configure the manipulation of articulated robots which moved similarly to humans. RULA was used to analyse the coincidence between the robot and the human [110]. Continuing in the field of robotics, one study focused on the design and analysis of a system based on gesture control. The goal was to allow workers to control an industrial-type robot. The RULA [62] method was also used for its design.

Moreover, RULA was combined with other advanced tools to carry out new research. To improve industrial production, an ergonomic study was combined with virtual reality. The design of a cell was carried out to reduce worker fatigue [54]. Conversely, RULA was combined with Kinect to collect information on the movements made by workers during mounting and assembly tasks [72,103]. Likewise, an ergonomic assessment was performed using the DELMIA (Digital Enterprise Manufacturing Interactive Application) simulation tool for assembly tasks in the robotics industry. RULA determined the harmful postures and the necessary redesign of the work to improve them [85]. Finally, a system was developed using sensors placed on the workers’ bodies. RULA was used by a computer in real time, displaying the results on a screen. When these were very harmful, acoustic and visual signals were initiated. It was concluded that this system, which showed the information to the worker, reduced the occurrence of MSD [101].

Rivero et al. [89] carried out an assessment of industrial and construction workers using RULA. They explained that increased productivity is closely linked to good worker ergonomics. Other authors also used it in conjunction with OSHA and the NIOSH equation. They concluded that rapid changes were required in 65% of the industrial tasks [84,94].

Baptista et al. [102] conducted three studies with three methods, including RULA. In total, 109 factory workers participated. The results showed that there were workers suffering from MSD, although no indications were presented. Finally, Yazdanirad et al. [61] also used three assessment methods to assess 210 people in three industries (the automotive, pharmaceutical and assembly industries). The results concluded that RULA was the most suitable method.

#### 3.1.4. Water Supply; Sewerage, Waste Management and Remediation Activities

Table 6 presents a single study belonging to this category.

Cakit [128] conducted a study to analyse waste collection workers. A programme was used to assess the postures adopted during loading and unloading. In addition, the RULA and REBA methods were used. They concluded the need for urgent changes in the postures adopted.

#### 3.1.5. Construction

Table 7 presents studies conducted in the construction field using RULA.

Shanahan et al. [132] analysed the usefulness of three assessment methods (RULA, REBA and the Strain Index) when used in non-fixed tasks and comparing them with four psychophysical scales from Borg 10. Fourteen construction workers without MSD were followed up. They concluded that the best method for this type of task was the Strain Index.

Another study proposed a more accurate way of measuring the angles in the postures adopted by construction workers in order to use the RULA method [131].

Finally, Li et al. [129,130] also focused on the field of construction. They developed a 3D system that allows one to simulate the working environment and perform an ergonomic assessment. They also applied REBA and RULA for the analysis.

#### 3.1.6. Wholesale and Retail Trade; Repair of Motor Vehicles and Motorcycles

In this category, only three studies are presented (Table 8).

##### Wholesale and Retail Trade and Repair of Motor Vehicle and Motorcycles

Risk factors and the onset of MSD were studied in car repair mechanics. In total, 191 workers participated. The modified Nordic Questionnaire was used with RULA, in addition to other tools. Most workers were shown to have this type of disorder [133].

##### Retail Trade, Except Motor Vehicles and Motorcycles

Capodaglio [134] conducted an ergonomic study on 70 clothing store vendors. RULA, REBA, the Strain Index and OCRA were used. The results showed a high risk in the upper extremities for the postures adopted. Another study was carried out at supermarket check-outs. A tool was developed to carry out a postural assessment of the workers. It was a portable and wireless system based on RULA and the Strain Index. It allowed the assessment to be performed as the task was being undertaken [135].

#### 3.1.7. Transportation and Storage

Table 9 contains the studies available in the Transportation and Storage category.

##### Land Transport and Transport via Pipelines

The RULA method was used in various studies to analyse drivers of different vehicles, such as garbage trucks and road-cleaning trucks. In total, 77 workers participated. The study concluded that the neck was the most affected area. The scores obtained varied depending on whether an adjustable seat was used [147]. Drivers of dangerous goods vehicles also suffered discomfort in the neck region, as well as in the feet, ankles, hands, etc. According to RULA, immediate changes to the tasks were required [136]. Similarly, car drivers were assessed, but this time RULA was used along with other methods such as OWAS and CORLETT. Goniometry was applied on some workers. The results showed different levels of discomfort in various parts of the body [145]. The method was used together with OCRA to assess workers in five transport company posts. In addition, a group interview was carried out. RULA demonstrated action Levels 1 and 2 [143].

Hoy et al. [146] studied the vibrations and postures to which forklift drivers were exposed. RULA, OWAS and a questionnaire were applied, and the vibrations were measured. The posture test results showed frequent lumbar discomfort. The highest risk level postures were those comprising a bent or twisted torso.

Another such study focused on wheelchair-support bus drivers. The assessment involved four workers and one passenger. Three different wheelchairs were used. The RULA, REBA, PLIBEL and iLMM methods were employed. High-level risks were determined during this task [144].

Balaji and Alphin [140] focused on drivers of industrial vehicles. Their objective was to optimise the worker’s area in the vehicle in order to reduce discomfort from the postures adopted. The software analysis was performed using RULA and REBA. Nearly half of the workers performed the task at a high-risk level. The areas most affected were the arm, wrist and torso.

Lastly, a new type of nozzle was developed for fuel refilling hoses. One hundred people were assessed using RULA while putting fuel in their vehicles. The postures were shown to be less harmful with the new design [139].

##### Warehousing and Support Activities for Transportation

In total, 92 workers from a supermarket warehouse were ergonomically assessed, specifically in two tasks—lifting boxes and pulling them. RULA was used in conjunction with other tools. Lifting the boxes affected the workers’ lower back, while the other task affected the lower arm and wrist [142]. Using the same method in the same workplace, they assessed the effect caused by a new storage tool. They concluded that it allowed for better work completion and resulted in less back and shoulder risks [141].

Finally, material handling workers were assessed. Sensors capable of measuring angles during a work task were used and, from these data, RULA was applied. The results showed high levels of risk in the postures adopted [137].

##### Air Transport

Some authors performed a study in which a new RULA method was developed. This allowed astronauts to be posturally assessed [138].

#### 3.1.8. Accommodation and Food Service Activities

Three studies are differentiated in this field of knowledge (Table 10).

Workers in an industrial kitchen, chefs at a Chinese restaurant and pub workers were assessed. RULA was used in all of these along with other ergonomic methods or tools. In the first case, RULA obtained more harmful results than for the other methods applied. The second showed that repetitive actions were performed on the upper extremities. The third concluded that very harmful tasks existed that required corrective actions [148,149,150].

#### 3.1.9. Information and Communication

Table 11 is composed of the different studies that have been carried out in the area of Information and Communication.

Several studies have been carried out on people who work regularly on a computer. One of these studies investigated whether the position where the mouse was placed with respect to the keyboard influenced the arm and shoulder postures adopted. RULA and surface electromyography were used. Some results showed that the position taken by right-handed people was less damaging if the number keyboard was not used [159]. Another similar analysis was performed on 10 workers who were also visually impaired. Harmful postures were identified for all the participants, and several recommendations [156] were made. Ekinci et al. [151] carried out an assessment very similar to the previous cases, using other tools in addition to RULA. The objective was to check the effectiveness of pre-use ergonomic computer training. Ergonomic risk was shown to decrease as a result. One of the workers carrying out this same task was followed up. They indicated discomfort in the neck and upper right extremity. They received physiotherapy sessions and ergonomic intervention, after which RULA indicated an improvement in the results [154].

Other authors used RULA [158] to assess workers carrying out two specific tasks, one based on entering data using VDT (Visual Display Terminal) screens and another on document classification and sorting. The results showed that the first task required further assessment. The second had to be modified after identifying harmful postures. Another group of VDT workers had the ergonomic training they received assessed using the method. The results improved after this, reducing the action levels [153]. To this end, RULA was used in conjunction with the Visual Analog Scale (VAS) to test the benefits of ergonomic training and physiotherapy sessions. VDT operators from the Software Corporation participated. It was observed that the methods were beneficial as they decreased the risk factors and the development of musculoskeletal disorders [157].

Other authors ergonomically assessed workers at a technology company, focusing on programming, management, administration and marketing tasks. RULA deduced high action levels with no risk-free postures [155]. Lastly in this area, the RULA method was applied, along with a modified version of it (mRULA), on 29 people working in computer science. The goal was to know if mRULA was valid for ergonomic assessment. They concluded that it could be used, and that it was only necessary to make an observation [152].

#### 3.1.10. Financial and Insurance Activities

Table 12 encompasses a single research study.

An ergonomic study was carried out in the financial department of a hospital using the RULA method. The results showed the need for ergonomic actions. This could reduce the grounds for absenteeism due to work-related accidents and increase well-being at work [160].

#### 3.1.11. Professional, Scientific and Technical Activities

Publications relating to this category are presented below (Table 13).

##### Other Professional, Scientific and Technical Activities

A study was carried out to establish a procedure for product design managers to perform an ergonomic assessment at the start of the design. This was developed for work carried out on desktops. To this end, information on the movement of workers was collected and a digital human model was created. Software incorporating the RULA method was used to perform the ergonomic analysis [162].

##### Veterinary Activities

The work of 12 veterinarians was assessed while performing four different tasks. Several tools were used, including a modification of RULA, to assess the wrist. It was concluded that, of the four tasks, only the posture adopted for suturing was not detrimental to the wrist [161].

#### 3.1.12. Administrative and Support Service Activities

In this field of knowledge, 12 studies have been analysed (Table 14).

##### Building Services and Landscape Activities

Two studies were carried out on cleaning workers using the RULA method. The first was used in conjunction with the REBA method and the Standardized Nordic Questionnaire. The results indicated risks ranging from medium to very high [166]. The second also made use of the Manual Task Risk Assessment (ManTRA) and QEC and the cleaning task was assessed. The results showed that the staff developed musculoskeletal disorders [172].

##### Office Administration, Office Support and Other Business Support Activities

Several research studies were conducted on the risks of MSD for office workers. One of these focused on computer staff. The workers were divided into two groups, one of which contained workers already suffering from discomfort. The ROSA (Rapid Office Strain Assessment), RULA and Maastricht questionnaire methods were used. RULA indicated worse scores for workers suffering discomfort [165]. Two similar studies were performed (one of which was determined with RULA) showing that more than half the workers suffered from musculoskeletal disorders and that the most injured areas of the body were the back and upper limbs. The other study proposed corrective actions for the work carried out [169,174]. Administrative workers were ergonomically studied in the same way. In addition to using RULA, measurements were taken of the workers and the office furniture. The study concluded that the subjects were developing MSD. Corrective actions were necessary over the short term [170].

Two further studies were carried out with RULA using an intervention group and a control group. In one of them, two groups were created containing 100 workers, and lighting data and information were collected. It was determined that the use of adjustable lights improved both the ergonomics and vision while the tasks were carried out [167]. In the other study, the intervention group used a particular keyboard, mouse and touchscreen. RULA showed a decrease in harmful upper limb postures although this worsened for the hands [168].

Several works were also carried out, the objective of which was to assess ergonomic programmes. One of them was performed in Iran on office workers in a petrochemical business. A questionnaire and the RULA method were used for the assessment. Improvements were made to the postures affecting the neck, shoulders, upper back and lumbar area thanks to the ergonomic programme [163]. With the same purpose in mind, Taieb-Maimon et al. [173] assessed three groups of workers who used computers in offices, one the control group and two that had received different types of training. Workers were assessed with RULA before, during and after the training. It was observed that the two groups with training improved their postures in a short period of time. Dalkilinc and Kayihan [171] also demonstrated that ergonomic training improved the postures adopted by office workers; they used RULA and surveys. In this case, it was web training.

Finally, in this field of knowledge, Cavalini et al. [164] verified an upper extremity assessment questionnaire called the Upper Extremity Work Demand (UEWD-R). The assessment was carried out using RULA with the help of office workers who used computers. The results indicated that the questionnaire was valid for assessing postural load on upper extremities.

#### 3.1.13. Public Administration and Defence; Compulsory Social Security

Table 15 presents the research developed in this field.

Nam et al. [175] assessed the postural load on soldiers when cleaning cannons in two different ways: manually and with an automated tool. OWAS, RULA and REBA were applied. They concluded that there was a lower risk (Level 2) when using the tool than with manual cleaning (Level 4). Another assessment of this type was developed for firefighters and emergency physicians. RULA, REBA and the NIOSH equation were used. The results showed high risks, indicating the need for corrective action [176].

#### 3.1.14. Education

Table 16 consists of 12 studies developed in education.

Breen et al. [188] and Kelly et al. [187] used RULA and other tools to study the postures adopted by students on the computer. Some authors focused on primary schools and others on high schools. They concluded that about 60% of the postures belonged to action Level 2. Sellschop et al. [177,179] provided ergonomic training to students with the same characteristics. They assessed effectiveness using two groups (the intervention and control groups) with the same method and other tools. In the intervention group, the risk decreased according to RULA, with action Level 4 disappearing. Other authors found that the assessor’s experience of using RULA and task observation was not relevant. They demonstrated this by assessing children while they took part in ICT. The study was performed by two groups of assessors, one of them experienced in applying the method [180].

Some authors [181,186] ergonomically analysed students at the university level, also while using computers. Two studies are differentiated, one using the RULA method and the other a modification of it (mRULA) in addition to other tools. It was shown that, at an engineering university, 30.8% of participants were exposed to a Level 3 risk. In medicine, 42.9% were exposed to a Level 2 risk. On the other hand, when employed at the Qazyin University of Medical Sciences on workers using the computer, it was intended to assess a training programme. This was done in stages, through a control group and an intervention group. RULA, VAS and the Nordic Questionnaire were used. The intervention group improved. The programme proved beneficial to the workers [183].

Furthermore, in the field of education, other ergonomic analyses were carried out using RULA and other tools. They focused on students at different levels. In some cases, harmful postures were adopted due to, among other causes, the furniture used. New furniture designs were required [178,182]. Students aged between 13 and 15 were also assessed in a total of 104 positions. It was concluded that the 13-year-olds were more at risk [184].

Finally, other authors used a virtual environment to perform an ergonomic analysis on a folding bike. This was designed at the University of Indonesia. RULA was used along with other methods. The goal was to obtain the best design, which was completed when the handlebars were 32 cm high and the saddle at 83 cm [185].

#### 3.1.15. Human Health and Social Work Activities

Table 17 presents the studies according to the “Human health and social work activities” category.

This field is divided into different specialties to show the available studies in a more ordered fashion.

- Ophthalmology

Ratzlaff et al. [189] tested the usefulness of an ergonomic training course given to 10 ophthalmologists. They focused on a task that consisted of positioning a slit lamp. It was assessed before and after training, using RULA applied with software. Risk levels were shown to be lower after training.

- Radiology

In the United Kingdom, mammogram radiologists were ergonomically assessed. Three work posts were analysed, one for digital mammograms, one for film mammograms and the third for both. The RULA method was used. It was determined that no more MSDs developed as a result of performing the mammograms digitally [222].

- Nursing

Sezgin and Esin [207] evaluated 1,515 intensive care nurses. They mainly used the RULA method. They showed that the hardest-hit areas of the body were the back and legs. They also concluded that the scores for this method were lowered by giving the Ergonomic Risk Management Programme (ERMP) to workers [193].

Sung et al. [219] decided to use an armrest and mirror to observe the tubes after each blood test. These proposals reduced the RULA score from 7 to 2. Therefore, the postures of the workers improved, mainly in the neck and shoulders.

As a last study, Garosi et al. [191] designed and developed a new instrument for a nursing task that used to be done manually. According to RULA, the level of risk decreased from 3 to 2 in the upper extremities when using this new tool.

- Otolaryngology

Goyil et al. [192,199] analysed the postures adopted by the otolaryngologists when the patient was placed in position (sitting or lying on their back). The RULA method was used. Higher risk levels were determined for sitting patients.

- Ultrasounds

Workers performing ultrasounds were assessed because they often suffer from musculoskeletal discomfort. RULA was used on five participants and on 24 ultrasounds; other parameters were also taken into account. The results showed awkward postures for the upper limbs [213].

- Laboratory

Some authors found that lab workers in hospitals developed MSD from performing their tasks. In these studies, they applied RULA, sometimes with other methods or tools [204,212].

- Surgery

The use of robots in surgery does not always lead to correct postures in the workers. Several authors assessed surgical personnel during their work using RULA and other tools. They found detrimental results and the need to modify the tasks [190,215]. One solution could be to provide ergonomic training to workers who perform surgery with robots. According to RULA, this improved the levels of risk [195]. The use of robot in other cases led to ergonomic improvements. One of these was when performing endoscopies. Surgeons obtained lower RULA scores when using robots than when performing the operation manually [224].

Other authors focused their studies (using the RULA method) on surgeons performing laparoscopies. They showed that there was a risk regarding the postures the surgeons developed [197]. In some cases, they combined these studies with the use of new technologies. For example, Youssed et al. [218] used a virtual reality simulator in this type of study, whereas Sanchez-Margallo et al. [221] used a glove called a CyberGlove (R). This glove allowed one to measure the movements made by the wrist, which can then be applied to an adapted RULA method. Bensignor et al. [201] designed a robotic needle holder for this type of surgery. Using RULA, they demonstrated that surgeon’s postures were less harmful than when performing the technique in the traditional way.

Other ergonomic analyses were performed on surgeons carrying out various tasks. Li et al. [196] used RULA to assess plastic surgeons who used magnifying glasses, and who suffered discomfort in the upper extremities. They indicated that modifying the height of the table or the distance of the magnifying glass from the workplace could lessen these problems. Person et al. [225] used an optoelectronic system to take posture data and obtain results using a modified RULA. They found harmful results mainly at the wrist. Hermandon and Choi [217] agreed that the wrist was one of the hardest-hit areas. They used RULA and other tools on office-based surgery (OBS) workers. They concluded that these workers were at high risk of musculoskeletal disorders. In addition, they highlighted other areas of the body that were harmed such as the arms, shoulder, neck and back.

Other authors assessed surgeons’ postures using RULA when they performed the same procedure in different ways. For example, Statham et al. [220] focused on laryngeal microsurgery surgeons. They assessed them while performing the task adopting three different postures, one with the arms unsupported and the other two with different supports. They deduced the levels of risk to which the surgeons were exposed. Singh et al. [200] assessed gynaecology surgeons using four different chairs. They determined a medium-to-high risk in the neck and shoulders. They showed that the chairs did not influence the scores.

Finally, some authors presented the idea of integrating different ergonomic methods and tools to assess nursing and surgery workers. OWAS, REBA, RULA and NIOSH were used, amongst other methods [209].

- Dentistry

Several authors focused on ergonomically assessing dental students while they were doing their internships. This was done using RULA, sometimes together with other methods or questionnaires. They concluded that there was a high level of risk regarding musculoskeletal disorders [203,211,214]. Ergonomic improvements in this area showed that using a Bambach Saddle armchair resulted in lower risk than when using a conventional chair [223], and that using magnifying glasses was beneficial for adopting lower-risk postures [194].

This type of study was also conducted on dentists to assess their postures. RULA was used in conjunction with other methods in most cases. Marcon et al. [198] compared the difference between using a magnifying glass, a microscope or not using anything at all during operations. They showed that using a microscope led to more harmful postures. In addition, these dentists are usually harmed by the postures they adopt during their tasks. Therefore, it is necessary to establish changes, some of which are required immediately. The most affected parts of the body are the lumbar region and neck [206]. This coincides with that determined by Tirgar et al. [208], although they added that another area affected was the shoulders, as inferred by Rafie et al. [210].

To conclude this group, the ergonomics of dental hygienists were also studied. They used RULA, REBA and the Strain Index. It was concluded that the postures the hygienists adopted contributed to the onset of musculoskeletal disorders [216].

- Other

An ergonomic assessment of workers in an emergency pharmacy was carried out using three methods, including RULA. This led to some improvements in their work [205]. Another series of improvements was made for women workers in the biomedical field. An intervention was carried out based on changes to the workplace and recommendations. According to RULA, this was beneficial as it led to lower scores for more than half of the postures [226]. Finally, methods for analysing ergonomics in health-related jobs were studied. It was concluded that RULA could be used to attain more accurate results [202].

#### 3.1.16. Arts, Entertainment and Recreation

Table 18 includes only three studies.

In the field of music, some authors demonstrated, with RULA and other tools, that musicians often suffer MSD mainly in the upper extremities. The risk is higher in those who play string instruments [228]. The development of such disorders was also confirmed in potters, sculptors and craft workers in India. They made use of RULA and other assessment methods. For all cases, harmful postures were deduced as well as the need to make changes to avoid them, immediately for the crafts workers [227,229].

#### 3.1.17. Other Service Activities

Only three studies are presented in this category as well (Table 19).

##### Repair of Computers, and Personal and Household Goods

An ergonomic assessment was carried out on bicycle repair workers. The RULA, REBA and OCRA methods were used, along with other parameter measurement. The tasks assessed were shown to be at a high-risk level, and that immediate improvement actions were required [231].

##### Other Personal Service Activities

Some authors focused on spa workers, with the aim of preventing them from developing MSD during three of their tasks. An assessment was carried out with RULA and QEC. The results showed that ergonomic information and training would improve the postures adopted [232]. A similar study, using only RULA and a survey, was conducted on tattooists. It was concluded that 71% of the postures adopted had a high level of risk and therefore urgent corrective measures were needed [230].

#### 3.1.18. Activities of Households as Employers; Undifferentiated Goods- and Services-Producing Activities of Households for Own Use

Table 20 summarises the publications in this category.

Domestic workers are also exposed to the onset of musculoskeletal disorders, although, according to Apostoli et al. [234], at a medium or low level. This was deduced by research in which RULA and other assessment methods or tools were applied. Another similar study was carried out using the RULA and MAPFRE methods, but in this case the workers were in wheelchairs. It was inferred that there were risks caused by the postures that had to be adopted in the chair in order to carry out the tasks [233].

#### 3.1.19. Other Areas Not Previously Included

Finally, a category (Table 21) is presented that is not covered by the classification used but has applications that cannot be included in the above fields of knowledge.

The different studies available are divided into groups according to the topic covered:

- Sensors

The Kinect sensor has been used in several studies together with the RULA method for ergonomic analysis. Some of them have compared the results by performing the assessment without the sensor and were similar. With Kinect, you can reduce the assessment time or subjectivity with professional analysis [235,244].

- Use of mobile phones and computers

Some authors assessed people who sent SMS using their mobile phones. They used a questionnaire and the RULA method. The latter exposed a risk Level 3 for the right and left area of the body, so modifications [239] were required. Employing the same method, other authors assessed the postures of children using computers or other ICT devices. They deduced risk categories of Level 2 or higher for the latter. In the case of computers, it was inferred that the RULA method was not recommended for applying to girls under eight years of age [245,251].

- Methods

Can and Figlali [241] developed a new assessment method from RULA, called ARULA (Advanced RULA). This was characterised as improving the advantages of the initial method, such as its high pre-observation time, assessment, etc.

One assessment was carried out using various methods such as the State of Washington method, OCRA, RULA, the Strain Index, etc. A high or very high level of risk for the upper limbs [256] was obtained with all. Two of the above methods, the Strain Index and RULA, were also studied from the perspective of validity, reliability, etc. The analysis of various jobs that included static positions or repetitive tasks was carried out, concluding that training was necessary to apply the [236] methods. Finally, a study was conducted on perceived discomfort in the different postures of the upper extremities. The RULA method [248] was used.

- Technological Evolution

Technology was used to apply the RULA method in several studies. Manghisi et al. [242] developed software called K2RULA capable of assessing workers during the task or with subsequent analysis. Goncalves and Fernandes [253,255] developed a semi-automatic system for performing ergonomic assessments using the RULA method. It was based on two synchronised video recording cameras for observing the worker’s tasks. From these recordings, RULA automatically calculated the scores, obtaining the action level for each of the observations. Plantard et al. [246] developed a method for performing ergonomic assessments using a virtual mannequin and RULA scores. They noted that the results of this new method coincided with those that had actually been obtained. Finally, Schlette and Rossmann [252] adapted the RULA method with Virtual Human in order to control human movements. The goal was to control the movements of a humanoid robot that are similar to those performed by a human.

- Gestures

One study used RULA in conjunction with another method called Quick Rating (QRating) to ergonomically assess people who made typical gestures when communicating. Physical discomfort during these gestures should be controlled [243].

In another study, gestures were required for 18 commands used in the movement of objects in a virtual environment. To do this, they were determined using Korean sign language and others created by the user. All these gestures were analysed and the RULA method [250] was used for this purpose.

- Other works

Razavi and Behbudi [247] ergonomically analysed box office workers with the RULA method, following the adoption of a series of improvements. It was concluded that there was a reduced level of risk. The same type of study was carried out on workers at a gas bottle company. It determined the existence of postures with an action Level 3 risk, and the need for changes in a short period of time [249]. It was also carried out on workers who used machines and other equipment during their work. Anthropometric information and the OCRA method were also used [254].

On the other hand, Ward et al. [257] used RULA to assess the ergonomics of postures adopted in the use of biometric devices (fingerprints and veins on the palm of the hand). They concluded that the positions in which they were placed (angles and heights) influenced the postures adopted. McGorry and Lin [258] studied the influence on grip strength of the orientation and placement (height and distance) of tool handles. They used RULA to carry out an ergonomic assessment.

Finally, several authors developed two assessment methods, one that considered frequent and harmful postures and the other the time. An assessment was made, using these methods and a modified RULA method, of the postures adopted by a group of people. They deduced that there was no coincidence between the methods [259].

### 3.2. Country-by-Country Ranking

**Figure 5:**
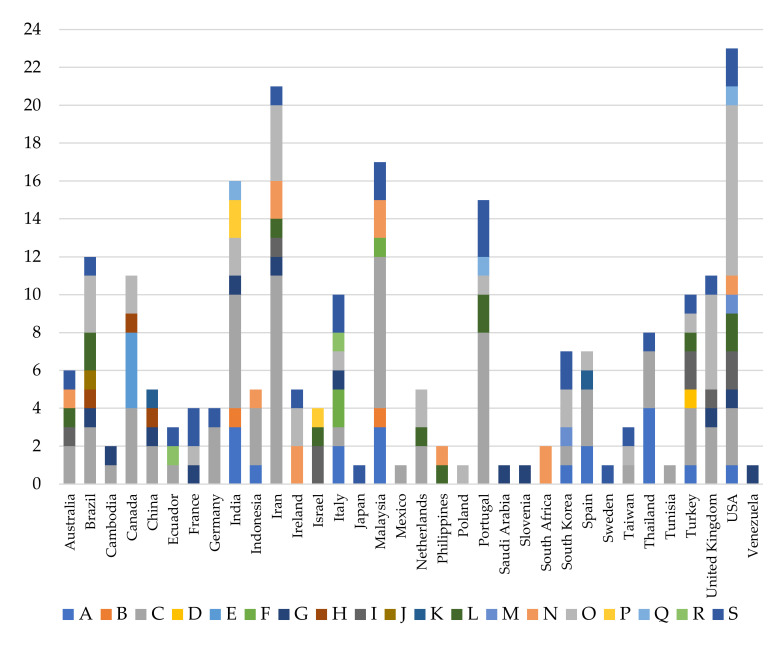
Number of studies classified by knowledge categories and countries: Agriculture, forestry and fishing (**A**); Mining and quarrying (**B**); Manufacturing (**C**); Water supply; sewerage, waste management and remediation activities (**D**); Construction (**E**); Wholesale and retail trade; repair of motor vehicles and motorcycles (**F**); Transportation and storage (**G**); Accommodation and food service activities (**H**); Information and communication (**I**); Financial and insurances activities (**J**); Professional, scientific and technical activities (**K**); Administrative and support service activities (**L**); Public administration and defiance; compulsory social security (**M**); Education (**N**); Human health and social work activities (**O**); Arts, entertainment and recreation (**P**); Other service activities (**Q**); Activities of households as employers; undifferentiated goods and services producing activities of households for own use (**R**); and Other scopes not previously included (**S**).

The revised applications are also classified according to their location, taking into account the fields of knowledge (Figure 5).

There are 34 countries where the RULA method has been used. The USA stands out with 23 studies, followed by Iran with 21, Malaysia with 17, India with 16, Portugal with 15, Brazil with 12 and Canada and the United Kingdom with 11. All other countries have 10 or fewer research studies. Eight locations have a single publication: Saudi Arabia, Japan, Mexico, Poland, Slovenia, Sweden, Tunisia and Venezuela (Figure 5).

In addition to the USA having the largest number of studies, it is also the country where the most knowledge categories have been covered. This comprises a total of 10 areas. “Human health and social work activities” stand out with nine studies, followed by “Manufacturing” with three (Figure 5). Both categories match the ones that have the most published studies at the general level (Figure 4 and Table 2).

The places following the USA are Turkey, Italy, Iran, India and Brazil, with seven different locations. In all of them, except Italy, the largest number of publications also corresponds to “Manufacturing”. Iran stands out with 11 studies in this category.

Countries such as Venezuela, Tunisia, Sweden South Africa, Slovenia, Poland, Mexico, Japan and Saudi Arabia only cover one area (Figure 5).

The United Kingdom is the location where the RULA method originated. However, it is not a place where it has been most applied, as only 11 studies have been performed there. The method originated with assessments carried out on workers in the industry [20]. Despite this, RULA has not been widely used in industry in the United Kingdom; it presents only three studies. “Human health and social work activities” stands out with five publications (Figure 5).

**Figure 6:**
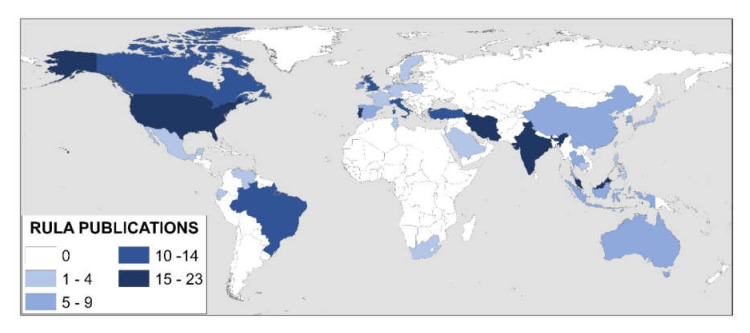
Map with frequencies.

Figure 6 shows the different countries according to the frequency of the published studies.

### 3.3. Classification by Years

**Figure 7:**
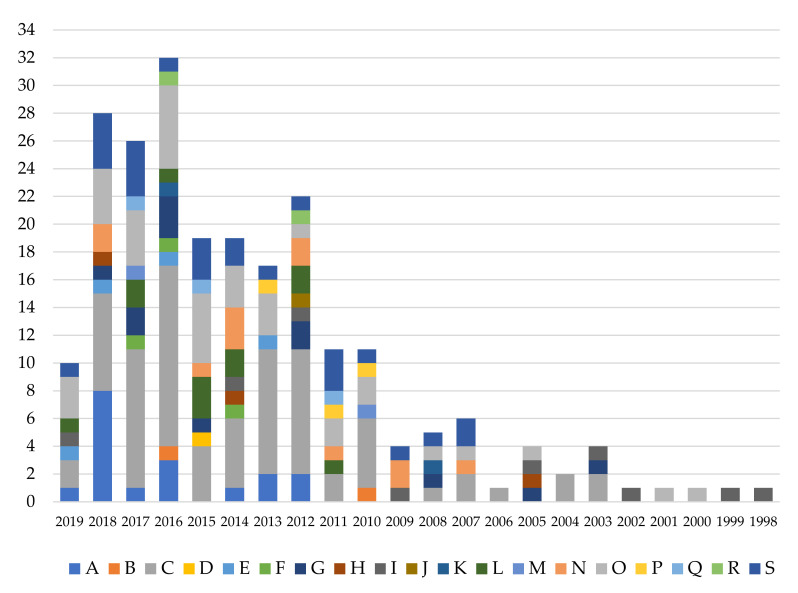
Number of studies classified by knowledge and year categories: Agriculture, forestry and fishing (**A**); Mining and quarrying (**B**); Manufacturing (**C**); Water supply; sewerage, waste management and remediation activities (**D**); Construction (**E**); Wholesale and retail trade; repair of motor vehicles and motorcycles (**F**); Transportation and storage (**G**); Accommodation and food service activities (**H**); Information and communication (**I**); Financial and insurances activities (**J**); Professional, scientific and technical activities (**K**); Administrative and support service activities (**L**); Public administration and defiance; compulsory social security (**M**); Education (**N**); Human health and social work activities (**O**); Arts, entertainment and recreation (**P**); Other service activities (**Q**); Activities of households as employers; undifferentiated goods and services producing activities of households for own use (**R**); and Other scopes not previously included (**S**).

Figure 7 shows the period of time in which RULA has been used and in which areas of knowledge.

The RULA method was created in 1993; however, according to the review carried out, it was not until 1998 that it was first applied.

From 1998 to 2019, the method has been applied every year. It should be noted that the year 2019 includes only the studies developed up until April.

The year 2016 stands out for the largest number of research studies (32). This is followed by the year 2018 with 28, 2017 with 26, 2012 with 22, 2014 and 2015 with 19 each, 2013 with 17 and 2010 and 2011 with 11 each. All other years have 10 or fewer studies.

Over the past 10 years, there has been an increase in such research. This may be due to the importance and awareness that occupational hazard prevention has received in recent times. In addition, it might be linked to the development of new technologies. These allow the method to be applied more quickly and efficiently (Figure 7).

It is noted (Figure 7) that over the past 10 years, the number of categories investigated has also increased compared to the previous 12 years. The year 2016 is characterised not only as having the largest number of publications, but also by the largest number of areas studied (11). “Manufacturing” stands out for representing 13 of the 32 research studies, followed by “Human health and social work activities” with 6. There are seven years with a single category (1998–2002, 2004 and 2006).

### 3.4. Classification by Journal

Table 22 presents all the journals available in this review, the number of articles published in each, knowledge categories (Web of Science), impact factor, rank and quartile. In total, 163 studies are differentiated. The rest of those that have been reviewed do not correspond to journal articles.

Of all the journals, there are four with a greater number of articles published: “Work—A Journal of Prevention Assessment & Rehabilitation” with 26 studies, followed by “Applied Ergonomics” with 16, “International Journal of Industrial Ergonomics” with 12 and “International Journal of Occupational Safety and Ergonomics” with 8 (Table 22).

### 3.5. Comparing Results with Other Methods

Some bibliographic reviews similar to this one have already been carried out. They have focused on other indirect and semi-direct assessment methods. Some matches are presented in the results obtained.

The RULA method matches the OWAS method and the Standardized Nordic Questionnaire in the most common knowledge categories. The field of industry and health and social assistance are the most researched, with one or the other being highlighted depending on the method [8,260]. The REBA method has also been highlighted in industry, although the health-related field ranks fourth [261].

For countries with the highest number of publications, none of the methods coincided with RULA, since REBA excelled in India, OWAS in Finland and the Nordic Questionnaire in Iran. However, for REBA, the second country with the most studies was the USA [8,260,261].

By date, the Nordic Questionnaire matches RULA. The year 2016 was when more studies were published for both [260].

Finally, with regards to journals, information is only available for the REBA method. The journal with the largest number of publications on this method is “Work—A Journal of Prevention Assessment & Rehabilitation”; this is also the case for RULA [261].

It should be noted that this comparison is general since the date range selected in each of the studies is not the same.

### 3.6. Sustainability and the RULA Method

**Figure 8:**
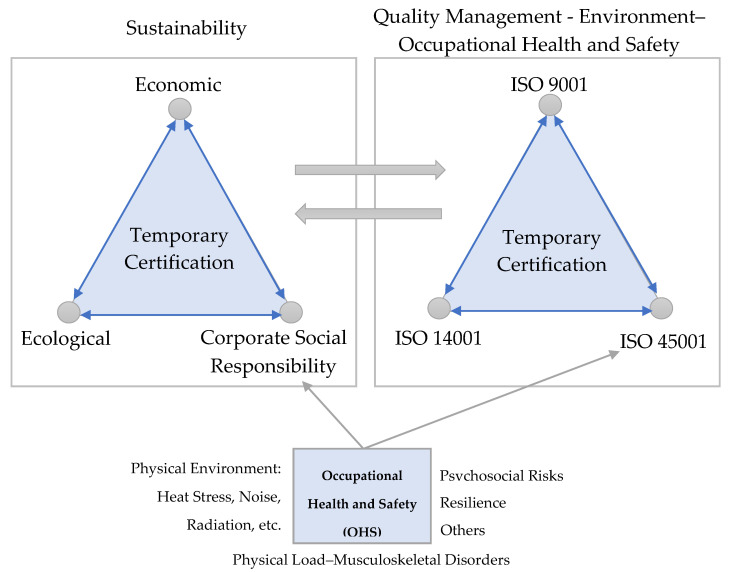
Parts of sustainability.

By integrating ISO 45001 with ISO 9001 and ISO 14001, organisations seek to implement quality management, occupational safety and health and the environment in a single system (Figure 8). The three systems, operating together, allow the organisation to optimise:

▪ The quality of the product or service
▪ Customer satisfaction
▪ The disposal of waste that has an impact on the environment
▪ The efficiency of the processes
▪ The health and safety of workers

Sustainability is a set of three fundamental parts: economic, social and environmental [262].

Sustainability, therefore, is not only focused on natural resources, but also plays a very important role in companies and contracted staff [263]. It fosters a relationship between healthy living and caring for nature [262]. For this reason, it is very important to also focus on the care of workers, carrying out assessments in order to obtain improvements in their health and safety [264].

The RULA method pursues this objective, as it focuses on the study of musculoskeletal disorders. Therefore, the use of this method contributes to the production of sustainable products.

### 3.7. Limitations

The country classification in this document has been based on the origin of the authors, as not all studies are clear about their location. The locations selected in each study have been those to which the greatest number of authors belong. In cases where it was not possible to follow this criterion, the location of the first author was selected.

## 4. Conclusions

The RULA method has been used in two main areas, “Manufacturing” and “Human health and social work activities”. However, it has been found not to be a sector-specific method. It can be applied to workers in any field.

Its use has been increasing over the last 10 years, perhaps indicating it is an effective method which is being tested more and more. This is also coupled with a growing awareness of workers’ health and safety. The year that stands out for the number of studies published is 2016 with a total of 32, followed by 2018 with a total of 28.

Countries such as the USA and Iran excel at applying the method and encompassing numerous fields of knowledge. The journal with the most publications is “Work—A Journal of Prevention Assessment & Rehabilitation”, with a total of 26. This journal is followed by “Applied Ergonomics” with 16 published studies.

RULA is usually used in combination with other ergonomic methods or tools to provide better and more reliable results. In this way, it is possible to contemplate different risk factors and evaluate numerous body areas. In addition, technological advancement is also appreciated in all sectors, since in many studies the method is combined with the use of sensors, software, virtual reality, etc.

## Figures and Tables

**Table 1:** Types of MSD assessment methods [8].

Direct	Semi-Direct	Indirect
Placing sensors on workers’ bodies as they perform tasks.	Observing the task being carried out and using software to analyse it.	Employing questionnaires.

**Table 2:** Knowledge categories [33].

Knowledge Category	Number of Studies
Agriculture, forestry and fishing	18
Mining and quarrying	2
Manufacturing	74
Water supply; sewerage, waste management and remediation activities	1
Construction	4
Wholesale and retail trade; repair of motor vehicles and motorcycles	3
Transportation and storage	12
Accommodation and food service activities	3
Information and communication	9
Financial and insurance activities	1
Professional, scientific and technical activities	2
Administrative and support service activities	12
Public administration and defence; compulsory social security	2
Education	12
Human health and social work activities	38
Arts, entertainment and recreation	3
Other service activities	3
Activities of households as employers; undifferentiated goods and services producing activities of households for own use	2
Other scopes not previously included *	25

* The category “Other areas not previously included” is not part of the classification used; it is a section that encompasses studies that have not been considered in the other fields.

**Table 3:** Agriculture, forestry and fishing.

Reference	Year	Country	Study Objective
[34]	2019	Italy	RULA and REBA assessment of a forestry worker using a wood-chipper machine.
[35]	2018	Thailand	Design of a knife to improve the ergonomics for workers who collect rubber. BCTQ and RULA were used for the assessment.
[36]	2018	USA	Use of RULA and other ergonomic tools on blueberry harvesters.
[37]	2018	India	Application of OWAS, RULA, REBA, QEC and others on rice growing workers.
[38]	2018	Thailand	Use of the Musculoskeletal Nordic Questionnaire, RULA and a tasks checklist in hazardous places for fruit farmers.
[39]	2018	India	Implementation of the modified Standardized Nordic Questionnaire and the RULA method on farm workers performing manual tasks.
[40]	2018	Spain	Use of RULA, REBA and OWAS on olive farm workers during the task of pruning with a chainsaw.
[41]	2018	India	Use of the Musculoskeletal Nordic Questionnaire and the RULA method on farm workers engaged in manual harvesting.
[42]	2018	South Korea	Comparison of the ALLA method with RULA, REBA and OWAS using the assessment of postures developed in agriculture.
[43]	2017	Turkey	Application of REBA, RULA, QEC and OWAS on nursery workers.
[44]	2016	Indonesia	RULA assessment on workers who use threshing machines for rice cultivation.
[45]	2016	Spain	Application of RULA on farm workers performing crop stringing
[46]	2016	Malaysia	Assessment using RULA and other ergonomic tools on workers responsible for cutting oil palm.
[47]	2014	Malaysia	Application of RULA on workers charged with oil palm collection.
[48]	2013	Malaysia	Use of RULA on oil palm harvesters.
[49]	2013	Thailand	Use of the Industrial Ergonomics Screening Tool, based on HAL (Hand Activity Level) and RULA methods on workers during rice cultivation ploughing with a cultivator.
[50]	2012	Italy	Conducting a study with the aim of redesigning the space for the driver of an agricultural tractor. Catia V5 software and other tools, including RULA, were used.
[51]	2012	Thailand	Use of a survey, a form and RULA on rubber harvesters.

**Table 4:** Mining and quarrying.

Reference	Year	Country	Study Objective
[52]	2010	India	Use of REBA, RULA, OCRA and other ergonomic tools on stone carving workers.
[53]	2016	Malaysia	Application of RULA on mine workers.

**Table 5:** Manufacturing.

Reference	Year	Country	Study Objective
[54]	2019	Turkey	The design of a cell in an industry using virtual reality and an ergonomic study with RULA.
[55]	2019	Malaysia	Application of RULA and interviews on workers in a car factory.
[56]	2018	India	Application of a questionnaire, RULA and REBA on brick kiln workers.
[57]	2018	Tunisia	Application of RULA and REBA on milling, turning and drilling workers.
[58]	2018	Canada	Use of the RULA method and certain devices on baristas responsible for tamping the espresso coffee.
[59]	2018	Iran	Application of a questionnaire and RULA on assembly workers in an electronic components factory.
[60]	2018	Malaysia	Use of the modified Nordic questionnaire, REBA and RULA on assembly workers in an electronics factory.
[61]	2018	Iran	Use of RULA, LUBA (Loading on the Upper Body Assessment) and NERPA (New Ergonomic Posture Assessment) on automotive, pharmaceutical and assembly workers.
[62]	2018	United Kingdom	Design and analysis of a system allowing workers to control an industrial robot. RULA was also used for its design.
[63]	2017	Malaysia	A car seat was designed with CATIA and then RULA was used for the ergonomic assessment.
[64]	2017	Iran	Use of RULA and another computer tool on sugar factory workers who manually moved the bags.
[65]	2017	Malaysia	Use of RULA and REBA on food industry workers.
[66]	2017	USA	Use of RULA and the Strain Index on aircraft factory workers.
[67]	2017	Italy	Immersive virtual reality for assembling an aircraft’s wing. RULA and REBA were used for the ergonomic assessment.
[68]	2017	United Kingdom	The development of a tool for the analysis of aircraft maintenance work. RULA, OWAS and LBA were employed for the ergonomic assessment.
[69]	2017	India	Use of RULA to assess pump-assembly workers who are submerged in wells.
[70]	2017	Ecuador	Application of OWAS and RULA on workers in a shoe factory.
[71]	2017	Iran	Use of the Nordic Questionnaire and RULA on workers in a pharmaceutical company.
[72]	2017	Australia	Use of Kinect and RULA on assembly workers.
[73]	2016	Brazil	Use of the Nordic Musculoskeletal Questionnaire and RULA on chemical industry workers.
[74]	2016	India	Design of a new crane cab in the steel industry. Ergonomic assessment with RULA.
[75]	2016	Portugal	RULA and the Strain Index were applied on 3 assembly posts manned by electronic production workers in the automotive industry.
[76]	2016	India	Use of an interview, questionnaire and RULA on workers who peel pineapples.
[77]	2016	Thailand	Use of RULA and other tools, such as the Nordic Questionnaire, on workers in a frozen food company.
[78]	2016	Iran	Use of interviews, the Nordic Musculoskeletal questionnaire and RULA on shoe sole production workers.
[79]	2016	Iran	Use of RULA and the Nordic Questionnaire in packaging workers in the pharmaceutical industry.
[80]	2016	Iran	Use of the Nordic Musculoskeletal Questionnaire and RULA on sewing workers.
[81]	2016	India	Application of RULA and questionnaires in textile industry workers.
[82]	2016	Indonesia	Use of RULA on workers in a batik cap factory.
[83]	2016	Cambodia	Conducting interviews and applying RULA to textile factory workers.
[84]	2016	Portugal	Use of OSHA, RULA and the NIOSH equation in industrial workers.
[85]	2016	China	Ergonomic assessment using DELMIA of assembly in the robotics industry. Using RULA.
[86]	2015	Portugal	Application of RULA on workers in charge of maintaining an oven.
[87]	2015	Iran	Use of a questionnaire and RULA on sewing machine workers.
[88]	2015	Indonesia	Study with RULA and the Nordic Body Map Questionnaire on industry workers tasked with producing batik caps.
[89]	2015	Mexico	Use of RULA on industrial and construction workers.
[90]	2014	Malaysia	Design of a new CNC milling machine. Ergonomic assessment of this machine, and the previous machine, using RULA.
[91]	2014	Iran	Use of a questionnaire and RULA on workers who sew shoes manually.
[92]	2014	Portugal	Application of RULA and the Nordic Musculoskeletal Questionnaire on footwear factory workers.
[93]	2014	Brazil	Use of a questionnaire and RULA on workers in a transformer factory.
[94]	2014	Portugal	Use of the OSHA Checklist, RULA and the NIOSH equation on factory workers.
[95]	2013	Spain	Use of RULA, REBA and virtual simulation in metal industry workers.
[96]	2013	Malaysia	Use of the RULA method and the Cornell Musculoskeletal Discomfort Questionnaire on manual handling workers in a metal stamping factory.
[97]	2013	Turkey	Use of a questionnaire and RULA on textile workers.
[98]	2013	Malaysia	Use of questionnaires based on the Standardized Nordic Questionnaire as well as the RULA and REBA methods on batik cap factory workers.
[99]	2013	Thailand	The ergonomic assessment of clothing assembly line workers using RULA.
[100]	2013	Malaysia	Application of RULA in packaging industry workers.
[101]	2013	Germany	Development of a system to ergonomically analyse workers in the industrial sector. The assessment was carried out using RULA.
[102]	2013	Portugal	Three different studies on factory workers, each using a method, which included RULA.
[103]	2013	Australia	The use of Kinect together with RULA on assembly workers.
[104]	2012	Brazil	Shoulder study on workers in a meat packing business. Using RULA, a human body diagram and anthropometric measurements.
[105]	2012	Taiwan	Study using 3D simulation to introduce humans into a robot workplace, working with fruits and vegetables. Ergonomic analysis using LBA and RULA.
[106]	2012	USA	Application of the RULA method and two other tools on mobile phone assembly workers.
[107]	2012	Netherlands	Ergonomic assessment with RULA on laptop manufacturing workers.
[108]	2012	Iran	Use of RULA and the Nordic Musculoskeletal Questionnaire on textile workers.
[109]	2012	Thailand	Application of REBA, RULA and OWAS on rubber sheet manufacturing workers.
[110]	2012	China	Development of a method to configure the manipulation of articulated robots. Using RULA.
[111]	2012	Canada	Use of 8 methods, including RULA, on industrial workers.
[112]	2012	Germany	Use of RULA to assess a humanoid robot arm with human-like postures.
[113]	2011	Turkey	Use of the adapted Nordic Musculoskeletal Questionnaire and RULA on textile workers.
[114]	2011	Indonesia	Use of a virtual space with a human model to ergonomically assess workers in a textile business. One of the methods used was RULA.
[115]	2010	Spain	Applying RULA through a Digital Human Model to assess roof slate manufacturing workers.
[116]	2010	Portugal	Use of RULA on cutlery polishing workers.
[117]	2010	Netherlands	Use of RULA and other tools on mobile phone assembly workers.
[118]	2010	USA	Use of 5 assessment methods, including RULA, on sawmill workers.
[119]	2010	India	Ergonomic assessment with RULA of assembly line workers.
[120]	2008	Canada	Use of various methods, including RULA, on sawmill workers.
[121]	2007	Canada	Use of 5 assessment methods, including RULA, on sawmill workers.
[122]	2007	South Korea	Use of REBA, RULA and OWAS on workers from various industries such as the electronics, steel, chemical and automotive industries.
[123]	2006	Portugal	Using RULA, Surface EMG, a questionnaire, and a clinical examination on paint workers in the automotive industry.
[124]	2004	Germany	Study of the benefits of employing a human digital model in the automotive industry and ergonomic assessment with RULA.
[125]	2004	Iran	Ergonomic analysis with RULA of workers in rug-fixing workshops.
[126]	2003	Spain	Application of RULA on workers in the metallurgical industry
[127]	2003	United Kingdom	Research to establish QEC action categories through the ergonomic assessment of industry workers, simultaneously applying QEC and RULA.

**Table 6:** Water supply; sewerage, waste management and remediation activities.

Reference	Year	Country	Study Objective
[128]	2015	Turkey	RULA and REBA assessment of workers in waste collection.

**Table 7:** Construction.

Reference	Year	Country	Study Objective
[129]	2019	Canada	Development of a 3D system to simulate the working environment in the construction field. Use of RULA in the ergonomic assessment.
[130]	2018	Canada	Development of a 3D visualisation tool for the construction field. Use of RULA in ergonomic assessment.
[131]	2016	Canada	Development of a new way to measure the angles that form the postures to apply RULA.
[132]	2013	Canada	Assessment with RULA, REBA and the Strain Index on construction workers.

**Table 8:** Wholesale and retail trade; repair of motor vehicles and motorcycles.

Reference	Year	Country	Study Objective
[133]	2014	Malaysia	Assessment using RULA, the modified Nordic Questionnaire and other tools on car repair mechanics.
[134]	2017	Italy	Assessment using RULA, REBA, the Strain Index and OCRA on clothing store vendors.
[135]	2016	Italy	Development of a portable and wireless tool based on the RULA method and the Strain Index for postural assessment.

**Table 9:** Transportation and storage.

Reference	Year	Country	Study Objective
[136]	2018	Brazil	Assessment with RULA and a survey on dangerous goods drivers.
[137]	2017	France	Application of RULA and measurement of angles with sensors in material handling workers.
[138]	2017	China	Development of a new method based on the RULA method.
[139]	2016	Iran	Development of a new nozzle for fuel hoses and assessment with RULA of the people who used it.
[140]	2016	India	Assessment with RULA, REBA and other tools on industrial vehicle drivers.
[141]	2016	Cambodia	Assessment of the use of a new tool using RULA for storage in supermarkets.
[142]	2015	Saudi Arabia	Application of RULA and other tools on supermarket warehouse workers.
[143]	2012	Venezuela	Assessment with RULA and OCRA of workers of a transport company.
[144]	2012	USA	Assessment with RULA, REBA, PLIBEL and iLMM of bus drivers responsible for handling wheelchairs.
[145]	2008	Slovenia	Application of goniometry and OWAS, RULA and CORLETT on car drivers.
[146]	2005	United Kingdom	RULA and OWAS were applied in addition to other tools on forklift drivers.
[147]	2003	Italy	RULA assessment of garbage truck and road cleaning drivers.

**Table 10:** Accommodation and food service activities.

Reference	Year	Country	Study Objective
[148]	2018	Brazil	Use of a survey and REBA, RULA and OWAS methods in industrial kitchen workers.
[149]	2014	China	Application of OWAS, RULA and the NIOSH equation in cooks at a Chinese restaurant.
[150]	2005	Canada	Use of 4 ergonomic tools, including RULA in pub workers.

**Table 11:** Information and communication.

Reference	Year	Country	Study Objective
[151]	2019	Turkey	Testing using RULA and other tools of the effectiveness of ergonomic training to people who make regular use of the computer.
[152]	2014	Israel	Application of the RULA method and a modification of it (mRULA) in computer workers, to check if mRULA was valid.
[153]	2012	Iran	Testing using RULA of whether there was an improvement or not in the ergonomic results in VDT workers after training.
[154]	2009	USA	Application of RULA to know the results of an ergonomic intervention in a worker who used the computer.
[155]	2005	Israel	Application of a questionnaire and RULA on workers of a technology company.
[156]	2003	United Kingdom	Use of RULA in workers who used the computer and were visually impaired.
[157]	2002	Turkey	The benefits of ergonomic training and physiotherapy sessions in VDT operators were studied. Use of RULA and other tools.
[158]	1999	USA	Use of RULA in workers who entered data with VDT screens and others that classified and sorted documents.
[159]	1998	Australia	Application of RULA and surface electromyography on computer workers.

**Table 12:** Financial and insurance activities.

Reference	Year	Country	Study Objective
[160]	2012	Brazil	Application of the RULA method on workers in the financial department of a hospital.

**Table 13:** Professional, scientific and technical activities.

Reference	Year	Country	Study Objective
[161]	2016	Spain	Application of OWAS and other tools to evaluate veterinarians.
[162]	2008	China	Ergonomic analysis using software based on the RULA method of people who perform their work on desktops.

**Table 14:** Administrative and support service activities.

Reference	Year	Country	Study Objective
[163]	2019	Iran	Assessment of the benefits of an ergonomic programme performed on office workers in a petrochemical company using a questionnaire and RULA.
[164]	2017	Netherlands	To verify an upper extremity assessment questionnaire, with the help of RULA.
[165]	2017	Brazil	Application of ROSA, RULA and the Maastricht questionnaire to assess office workers.
[166]	2016	Philippines	Use of the Standardized Nordic Questionnaire, and the RULA and REBA methods on cleaning workers.
[167]	2015	USA	Application of RULA and a lighting study on office workers.
[168]	2015	USA	RULA was applied, among other tools, to office workers who used a computer.
[169]	2015	Brazil	RULA, along with other methods and questionnaires, was applied to office workers.
[170]	2014	Portugal	RULA was applied to administrative workers.
[171]	2014	Turkey	The effectiveness of web ergonomic training for office workers was assessed. The RULA method and surveys were used.
[172]	2012	Australia	Use of ManTRA, QEC and RULA on cleaning workers.
[173]	2012	Israel	Application of RULA on office workers who use computers in order to assess the usefulness of a training method.
[174]	2011	Portugal	The RULA method was applied to office workers.

**Table 15:** Public administration and defence; compulsory social security.

Reference	Year	Country	Study Objective
[175]	2017	South Korea	Application of OWAS, RULA and REBA on soldiers.
[176]	2010	USA	Application of REBA, RULA and the NIOSH equation on firefighters and emergency physicians.

**Table 16:** Education.

Reference	Year	Country	Study Objective
[177]	2018	South Africa	Use of RULA and a questionnaire on students using the computer.
[178]	2018	Philippines	Application of RULA and REBA on students in a chemistry laboratory.
[179]	2015	South Africa	Use of RULA and VAS to assess ergonomic training carried out with school students who used a computer.
[180]	2014	Australia	Ergonomic assessment with RULA of children using ICT (Information and Communications Technology) to ascertain if the assessor’s experience is relevant in this method.
[181]	2014	Iran	Use of the Nordic Musculoskeletal Questionnaire, the RULA method and other methods as well as interviews on people who used computers at two universities.
[182]	2014	Malaysia	The use of RULA and other tools on elementary school students.
[183]	2012	Iran	Assessing a training programme using RULA and other tools on university workers using computers.
[184]	2012	Malaysia	Application of a questionnaire, REBA and RULA on students between 13 and 15 years old.
[185]	2011	Indonesia	Use of a virtual environment for ergonomic analysis of a bicycle. PEI was used, containing methods such as RULA.
[186]	2009	USA	Application of the modified RULA method and the University of California Computer Use Checklist on university students using computers.
[187]	2009	Ireland	The RULA method and other tools were used on high school students using computers.
[188]	2007	Ireland	Application of RULA, the body discomfort chart and VAS on school children using computers.

**Table 17:** Human health and social work activities.

Reference	Year	Country	Study Objective
[189]	2019	Canada	To check the usefulness of a training course for ophthalmologists on the placement of a slit lamp, using RULA.
[190]	2019	Netherlands	Use of RULA, among other tools, on operating room nurses using the da Vinci robot.
[191]	2019	Iran	Assessment using RULA of workers performing a task in a traditional way and with a newly designed instrument.
[192]	2018	USA	Application of RULA on otolaryngologists.
[193]	2018	Ireland	Use of RULA and other tools on ICU nurses.
[194]	2018	United Kingdom	Use of RULA and statistical analysis on dental students.
[195]	2018	Netherlands	To check if it was effective to give ergonomic training to surgical personnel in using robots, performed with RULA and other tools.
[196]	2017	United Kingdom	Ergonomic assessment with RULA on plastic surgeons who used magnifying glasses.
[197]	2017	India	Postural assessment of surgeons during a laparoscopic operation.
[198]	2017	Italy	Use of RULA and another method on dentists while carrying out an operation.
[199]	2017	USA	The RULA method was used to assess postures adopted by otolaryngologists.
[200]	2016	USA	Ergonomic assessment (including the RULA method) on gynaecology surgeons using 4 different chairs and several tools.
[201]	2016	France	Use of RULA together with a tool to assess the use of a new instrument designed for use by surgeons.
[202]	2016	United Kingdom	Study on the methods used, such as RULA, to analyse ergonomics in health-related jobs.
[203]	2016	Iran	Ergonomic assessment of dental students using RULA and a questionnaire.
[204]	2016	Portugal	Ergonomic study of workers in a hospital laboratory, using RULA.
[205]	2016	Brazil	Ergonomic assessment using 3 methods, including RULA, of emergency pharmacy workers.
[206]	2015	South Korea	Application of RULA and QEC on dentists.
[207]	2015	Turkey	Use of a form and RULA on intensive care nurses.
[208]	2015	Iran	Use of the RULA method and other tools on dentists.
[209]	2015	Poland	Integration of different ergonomic methods or tools, such as RULA, in nursing and surgery workers.
[210]	2015	Iran	Use of a questionnaire, RULA and the Nordic Musculoskeletal Questionnaire on dentists.
[211]	2014	Brazil	Use of RULA to assess upper limb postures in dental students.
[212]	2014	India	Use of RULA and other ergonomic tools on laboratory technicians.
[213]	2014	USA	Ergonomic study using RULA on workers performing ultrasounds.
[214]	2013	Brazil	Use of OWAS and RULA on students in the last year of dental studies.
[215]	2013	USA	Ergonomic study using RULA and other ergonomic tools on surgeons manipulating a robot.
[216]	2013	South Korea	Use of REBA, RULA and the Strain Index on dental hygienists.
[217]	2012	USA	Ergonomic analysis using RULA, amongst other tools, on office-based surgery workers.
[218]	2011	USA	Using a virtual reality simulator on surgeons performing a laparoscopic cholecystectomy. Ergonomic assessment with RULA.
[219]	2011	Taiwan	RULA assessment of one of the tasks performed during blood tests.
[220]	2010	USA	Application of RULA and three-dimensional imaging on surgeons who perform laryngeal microsurgery.
[221]	2010	Spain	Data collection using CyberGlove (R) and assessment using an adapted RULA method.
[222]	2008	United Kingdom	Application of the RULA method on mammogram radiologists.
[223]	2007	United Kingdom	Ergonomic assessment using RULA on dental students using two different armchairs.
[224]	2005	USA	Use of the Job Strain Index and RULA to assess surgeons performing an endoscopy manually and with the help of a robot.
[225]	2001	Canada	Use of an optoelectronic system to collect posture data on surgeons, and then assessing them with a modified RULA.
[226]	2000	Ireland	Ergonomic intervention in biomedical workers. RULA was used in the ergonomic assessment, among others.

**Table 18:** Arts, entertainment and recreation.

Reference	Year	Country	Study Objective
[227]	2013	India	The modified Nordic Questionnaire, REBA and RULA were applied to potters and sculptors.
[228]	2011	Israel	RULA and questionnaires were used on orchestral musicians.
[229]	2010	India	RULA, REBA, OVAKO, OCRA and the Strain Index were applied to craft workers.

**Table 19:** Other service activities.

Reference	Year	Country	Study Objective
[230]	2017	USA	Use of a survey and the RULA method on tattooists.
[231]	2015	India	REBA, RULA and OCRA were used on bicycle repair workers.
[232]	2011	Portugal	An ergonomic study using RULA and QEC was conducted on workers from four spas.

**Table 20:** Activities of households as employers; undifferentiated goods-and services-producing activities of households for own use.

Reference	Year	Country	Study Objective
[233]	2016	Ecuador	Assessment of people in wheelchairs who performed some domestic or work activity, using the RULA method and MAPFRE.
[234]	2012	Italy	5 ergonomic methods or tools were used, including RULA, on people performing household chores.

**Table 21:** Other scopes not previously included.

Reference	Year	Country	Study Objective
[235]	2019	Ecuador	New way to perform ergonomic analysis using the Kinect V2 sensor together with the RULA method.
[236]	2018	Brazil	RULA and the deformation rate (SI) were studied, and the analysis of various jobs was carried out.
[237]	2018	Malaysia	Conducting a study to assess the utility of the RULA-Kinect (TM) system.
[238]	2018	Malaysia	Research to compare the RULA method with the RULA-Kinect system.
[239]	2018	Thailand	Ergonomic study using a questionnaire and the RULA method on people who use mobile phones.
[240]	2017	France	A study using Kinect with the RULA method.
[241]	2017	Turkey	Development of the ARULA (Advanced RULA) method, which improves the advantages of RULA.
[242]	2017	Italy	Development of a software called K2RULA.
[243]	2017	South Korea	Use of RULA in conjunction with Quick Rating (QRating) to assess people making gestures while communicating.
[244]	2016	Australia	Using sensors, including Kinect for the RULA application.
[245]	2015	Sweden	Ergonomic assessment with RULA on children while they are using ICT devices.
[246]	2015	France	Development of a method for performing ergonomic assessments using a virtual mannequin and RULA scores.
[247]	2015	Iran	RULA employed on box office sales workers.
[248]	2014	Japan	Study of the relationship between upper limb discomfort and each degree of freedom. Using RULA.
[249]	2014	Taiwan	Use of RULA on workers at a gas bottle company.
[250]	2013	South Korea	Attempt to adopt gestures for virtual environment commands. RULA was used in the analysis.
[251]	2012	Ireland	Employing RULA on children who are computer users.
[252]	2011	Germany	Adapts the RULA method with Virtual Human to control human movements.
[253]	2011	Portugal	Development of a semi-automatic system with two video cameras and RULA.
[254]	2011	Portugal	Application of RULA and OCRA on workers using machines and other equipment.
[255]	2010	Portugal	Development of a semi-automatic system for ergonomic assessment using RULA and two synchronised cameras.
[256]	2009	Italy	Assessment performed with several methods, including RULA.
[257]	2008	United Kingdom	Study of two biometric-type devices. RULA was used.
[258]	2007	USA	Influence of orientation and placement of tool handles on gripping force. RULA was used.
[259]	2007	USA	Two methods were developed and a modified RULA method was also used.

**Table 22:** Number of publications (N) by scientific journal, knowledge categories (Web of Science), impact factor, rank and quartile (2018).

Journal	N	Impact Factor	Categories	Rank	Quartile
Advanced Science Letters	1	1.253	Multidisciplinary Sciences—SCIE	15/59	Q2
Agronomy	1	2.259	Agronomy—SCIE	19/89	Q1
			Plant Sciences—SCIE	78/228	Q2
American Journal of Obstetrics and Gynecology	1	6.120	Obstetrics & Gynecology SCIE	2/83	Q1
American Journal of Veterinary Research	1	1.070	Veterinary Sciences—SCIE	65/141	Q2
Applied Ergonomics	16	2.610	Ergonomics—SSCI	3/16	Q1
			Psychology, applied—SSCI	25/82	Q2
			Engineering, industrial—SCIE	20/46	Q2
Archives of Environmental & Occupational Health	1	1.483	Environmental Sciences—SCIE	181/251	Q3
			Public, environmental & occupational health—SCIE	124/186	Q3
Automation in Construction	1	4.313	Construction & Building Technology—SCIE	8/63	Q1
			Engineering, Civil—SCIE	7/132	Q1
Balkan Medical Journal	1	1.203	Medicine, General & Internal—SCIE	94/160	Q3
Behaviour & Information Technology	1	1.429	Computer Science, Cybernetics—SCIE	13/23	Q3
			Ergonomics—SSCI	8/16	Q2
Brazilian Journal of Physical Therapy	1	1.879	Orthopedics—SCIE	38/76	Q2
			Rehabilitation—SCIE	27/65	Q2
British Dental Journal	2	1.438	Dentistry, Oral Surgery & Medicine—SCIE	59/91	Q3
British Journal of Biomedical Science	1	2.365	Medical Laboratory Technology—SCIE	12/29	Q2
Canadian Journal of Civil Engineering	1	0.742	Engineering, Civil—SCIE	111/132	Q4
Canadian Journal of Ophthalmology—Journal Canadien D Ophtalmologie	1	1.305	Ophthalmology—SCIE	49/60	Q4
Ciencia UNEMI	1	No impact factor.			
Clinical Biomechanics	1	1.977	Engineering, Biomedical—SCIE	53/80	Q3
			Orthopedics—SCIE	35/76	Q2
			Sport Sciences—SCIE	39/83	Q2
Cogent Engineering	1	No impact factor.			
Collegium Antropologicum	1	0.609	Anthropology—SSCI	48/82	Q3
Design Journal	1	No impact factor.			
DYNA-Colombia	1	No impact factor.			
Ergonomics	7	2.181	Engineering, industrial—SCIE	21/46	Q2
			Ergonomics—SSCI	5/16	Q2
			Psychology—SCIE	38/77	Q2
			Psychology, applied—SSCI	35/82	Q2
European Journal of Dental Education	1	1.531	Dentistry, Oral Surgery & Medicine—SCIE	48/91	Q3
			Education, Scientific Disciplines—SCIE	23/41	Q3
Fresenius Environmental Bulletin	1	0.691	Environmental Sciences—SCIE	240/251	Q4
Global Nest Journal	1	0.869	Environmental sciences—SCIE	232/251	Q4
Health Promotion Perspectives	1	No impact factor.			
Health Scope	2	No impact factor.			
Human Factors and Ergonomics in Manufacture & Service Industries	2	1.000	Ergonomics—SSCI	13/16	Q4
			Engineering, manufacturing—SCIE	45/49	Q4
Independent Journal of Management & Production	1	No impact factor.			
Indian Journal of Community Health	1	No impact factor.			
Indian Journal of Occupational and Environmental Medicine	3	No impact factor.			
Industrial Health	1	1.319	Environmental Sciences—SCIE	201/251	Q4
			Public, environmental & occupational health—SCIE	143/186	Q4
			Toxicology	86/93	Q4
Intelligent Decision Technologies-Netherlands	1	No impact factor.			
Intensive and Critical Care Nursing	1	1.652	Nursing—SSCI	30/118	Q2
			Nursing—SCIE	33/120	Q2
International Archives of Occupational and Environmental Health	1	2.025	Public, environmental & occupational health—SCIE	90/186	Q2
International Journal of Environmental Research and Public Health	1	2.468	Environmental Sciences—SCIE	112/251	Q2
			Public, environmental & occupational health—SSCI	38/164	Q1
			Public, environmental & occupational health—SCIE	67/186	Q2
International Journal of Industrial Engineering—Theory Applications and Practice	1	0.532	Engineering, industrial—SCIE	45/46	Q4
			Engineering, Manufacturing—SCIE	47/49	Q4
International Journal of Industrial Ergonomics	12	1.571	Ergonomics—SSCI	7/16	Q2
			Engineering, industrial—SCIE	28/46	Q3
International Journal of Injury Control and Safety Promotion	1	0.870	Public, environmental & occupational health—SSCI	146/164	Q4
International Journal of Integrated Engineering	1	No impact factor.			
International Journal of Interactive Design and Manufacturing—IJIDEM	1	No impact factor.			
International Journal of Occupational Safety and Ergonomics	8	1.377	Ergonomics—SSCI	9/16	Q3
			Public, environmental & occupational health—SSCI	110/164	Q3
International Journal of Productivity and Performance Management	1	No impact factor.			
International Journal of Technology	1	No impact factor.			
International Journal of Working Conditions	1	No impact factor.			
International Journal of Workplace Health Management	1	No impact factor.			
International Nursing Review	1	1.562	Nursing—SSCI	36/118	Q2
			Nursing—SCIE	38/120	Q2
Iranian Journal of Public Health	2	1.225	Public, environmental & occupational health—SSCI	122/164	Q3
			Public, environmental & occupational health—SCIE	149/186	Q4
Journal of Advanced Simulation in Science and Engineering	1	No impact factor.			
Journal of Agricultural Safety and Health	1	No impact factor.			
Journal of Back and Musculoskeletal Rehabilitation	4	0.814	Orthopedics—SCIE	65/76	Q4
			Rehabilitation—SCIE	60/65	Q4
Journal of Bionic Engineering	1	2.463	Engineering, Multidisciplinary—SCIE	28/88	Q2
			Materials Science, Biomaterials—SCIE	21/32	Q3
			Robotics—SCIE	13/26	Q2
Journal of Construction Engineering and Management	1	2.734	Construction & Building Technology—SCIE	15/63	Q1
			Engineering, civil—SCIE	32/132	Q1
			Engineering, industrial—SCIE	17/46	Q2
Journal of Engineering Science and Technology	1	No impact factor.			
Journal of Environmental and Public Health	1	No impact factor.			
Journal of Health and Safety at Work	1	No impact factor.			
Journal of Minimal Access Surgery	1	0.966	Surgery—SCIE	168/203	Q4
Journal of Minimally Invasive Gynecology	1	2.547	Obstetrics & Gynecology- SCIE	25/83	Q2
Journal of Musculoskeletal Pain	1	No impact factor.			
Journal of Occupational Health	2	1.800	Public, environmental & occupational health—SCIE	105/186	Q3
Journal of Physical Therapy Science	2	0.392	Rehabilitation—SCIE	61/64	Q4
Journal of Robotics	1	No impact factor.			
Journal of Robotic Surgery	1	No impact factor.			
Journal of Sound and Vibration	1	3.123	Acoustics—SCIE	5/31	Q1
			Engineering, Mechanical—SCIE	27/129	Q1
			Mechanics—SCIE	21/134	Q1
Journal of the Faculty of Engineering and Architecture of Gazi University	1	0.652	Engineering, multidisciplinary—SCIE	76/88	Q4
Laryngoscope	1	2.343	Medicine, research & experimental- SCIE	78/136	Q3
			Otorhinolaryngology—SCIE	12/42	Q2
Makara Journal of Technology	1	No impact factor.			
Medicina del Lavoro	2	0.583	Public, environmental & occupational health—SCIE	179/186	Q4
Occupational and Environmental Medicine	2	3.556	Public, environmental & occupational health—SCIE	1/186	Q1
Occupational Medicine-Oxford	1	1.222	Public, environmental & occupational health—SCIE	150/186	Q4
Otolaryngology-Head and Neck Surgery	1	2.310	Otorhinolaryngology—SCIE	13/42	Q2
			Surgery—SCIE	72/203	Q2
Otology & Neurotology	1	2.063	Clinical Neurology—SCIE	134/199	Q3
			Otorhinolaryngology—SCIE	15/42	Q2
Pain Clinic	1	No impact factor.			
Physical Therapy	1	3.043	Orthopedics—SCIE	16/76	Q1
			Rehabilitation—SCIE	7/65	Q1
PLOS ONE	2	2.776	Multidisciplinary Sciences—SCIE	24/69	Q2
Safety and Health at Work	1	1.431	Public, environmental & occupational health—SSCI	102/164	Q3
			Public, environmental & occupational health—SCIE	131/186	Q3
Safety Science	1	3.619	Engineering, industrial—SCIE	10/46	Q1
			Operations research & management science—SCIE	16/84	Q1
Sensors	1	3.031	Chemistry, Analytical—SCIE	23/84	Q2
			Electrochemistry—SCIE	12/26	Q2
			Instruments & Instrumentation—SCIE	15/61	Q1
South African Journal of Physiotherapy	1	No impact factor.			
Surgical Endoscopy and Other Interventional Techniques	4	3.209	Surgery—SCIE	39/203	Q1
Surgical Endoscopy—Ultrasound and Interventional Techniques	1	No impact factor.			
Turkish Journal of Physical Medicine and Rehabilitation	1	0.223	Rehabilitation—SCIE	65/65	Q4
Work—A Journal of Prevention Assessment & Rehabilitation	26 *	1.009	Public, environmental & occupational health—SSCI	138/164	Q4
Workplace Health & Safety	1	0.922	Nursing—SSCI	91/118	Q4
			Nursing—SCIE	93/120	Q4

* Mode.

## Appendix A

The following table shows a summary of the meanings of the abbreviations.

**Table A1:** Abbreviations.

Abbreviations	Meaning
ALLA	Agricultural Lower Limb Assessment
BCTQ	Boston Carpal Tunnel Syndrome Questionnaire
CMDQ	Cornell Musculoskeletal Discomfort Questionnaire
CTD	Cumulative Trauma Disorders
DELMIA	Digital Enterprise Manufacturing Interactive Application
EMG	Electromyograpgy
FECYT	Spanish Foundation for Science and Technology
HAL	Hand Activity Level
IBV	Instituto de Biomecánica de Valencia (In Spanish)
ICT	Information and Communications Technology
iLMM	Industrial Lumbar Motion Monitor
INSHT	Instituto Nacional de Seguridad e Higiene en el Trabajo (In Spanish)
KIM	Key Indicator Method
LBA	Lower Back Analysis
LUBA	Loading on the Upper Body Assessment
MAC	Manual Handling Assessment Charts
ManTRA	Manual Task Risk Assessment
MSD	Musculoskeletal Disorders
NERPA	New Ergonomic Posture Assessment
NIOSH	National Institute of Occupational Safety and Health
NMSQ	Nordic Musculoskeletal Symptoms Questionnaire
OBS	Office-based Surgery
OCRA	Occupational Repetitive Action
OSHA	Occupational Safety & Health Administration
OWAS	Ovako Working Analysis System
PATH	Posture, Activity, Tools and Handling
PEI	Posture Evaluation Index
PLIBEL	Method for the identification of musculoskeletal stress factors which may have injurious effects
PRRI	Posture and Repetition Risk Factor Index
QEC	Quick Exposure Check
QRating	Quick Rating
REBA	Rapid Entire Body Assessment
ROSA	Rapid Office Strain Assessment
RULA	Rapid Upper Limb Assessment
SI	Strain Index
UEWD-R	Upper Extremity Work Demand
VAS	Visual Analog Scale
VDT	Visual Display Terminal
VIRA	Video film technique for Registration and Analysis of working postures and movements
WoS	Web of Science
